# Impact of second-line cetuximab-containing therapy in patients with KRAS wild-type metastatic colorectal cancer: results from the ITACa randomized clinical trial

**DOI:** 10.1038/s41598-017-11048-9

**Published:** 2017-09-05

**Authors:** Alessandro Passardi, Emanuela Scarpi, Fabio Gelsomino, Maria Angela Palladino, Andrea Casadei Gardini, Daniele Turci, Vincenzo Emanuele Chiuri, Claudia Mucciarini, Davide Tassinari, Angela Ragazzini, Giovanni Luca Frassineti, Martina Valgiusti, Paola Ulivi, Dino Amadori

**Affiliations:** 1 Department of Medical Oncology, Istituto Scientifico Romagnolo per lo Studio e la Cura dei Tumori (IRST) IRCCS, Meldola, Italy; 2 Unit of Biostatistics and Clinical Trials, Istituto Scientifico Romagnolo per lo Studio e la Cura dei Tumori (IRST) IRCCS, Meldola, Italy; 3 Oncology Unit, University Hospital of Modena and Reggio Emilia, Modena, Italy; 4 Medical Oncology Unit, Guglielmo da Saliceto Hospital, Piacenza, Italy; 5 Oncology Unit, S. Maria delle Croci Hospital, Ravenna, Italy; 6 Medical Oncology Unit, Vito Fazzi Hospital, Lecce, Italy; 7 Medical Oncology Unit, Ramazzini Hospital, Carpi, Italy; 8 Department of Oncology, Infermi Hospital, Rimini, Italy; 9 Bioscences Laboratory, Istituto Scientifico Romagnolo per lo Studio e la Cura dei Tumori (IRST) IRCCS, Meldola, Italy

## Abstract

The ITACa trial was designed to define the role of cetuximab (Cet) and bevacizumab (Bev) in combination with standard chemotherapy (CT, FOLFIRI or FOLFOX4) as first- and second-line treatment in metastatic colorectal cancer. All patients with WT KRAS tumors who had been enrolled in the first-line trial were randomized onto two independent second-line trials: CT or CT + Cet (study 2A) and CT + Bev or CT + Bev + Cet (study 2B). Patients with mutated KRAS were not eligible for randomization and were treated with CT alone (study 2A) or CT + Bev (study 2B). The primary endpoint was progression-free survival (PFS). 48 and 56 KRAS WT patients were randomized while 31 and 40 KRAS mutated patients were treated without randomization. Study 2A: median PFS was 3.4 (95%CI 2.3–4.6) and 6.2 (95%CI 4.3–7.8) months for the CT and CT + Cet arms, respectively, with a hazard ratio (HR) = 0.64 (95%CI 0.35–1.16, p = 0.144). Study 2B: median PFS was 7.7 (95%CI 4.1–10.1) and 4.9 (95%CI 3.2–7.0) months for CT + Bev and CT + Cet + Bev arms, respectively, with a HR = 1.31 (95%CI 0.76–2.26, p = 0.330). Notwithstanding limitations due to the small sample size, among patients with WT KRAS the addition of Cet to second-line CT increased PFS, whereas the addition of Cet to CT + Bev was associated with worse PFS.

## Introduction

Colorectal cancer (CRC) is the third most commonly diagnosed cancer among males and the second among females worldwide^1^. Combinations of fluorouracil (FU), leucovorin (LV) and oxaliplatin (FOLFOX), and of FU, LV, and irinotecan (FOLFIRI) are considered standard regimens for metastatic CRC (mCRC). Most patients with mCRC receive first-line chemotherapy (CT) and approximately 70% of those who progress after one line of CT will undergo at least one further line of systemic treatment^2–5^ consisting in CT in combination with molecular-targeted agents directed against epidermal growth factor receptor (EGFR) or vascular endothelial growth factor (VEGF).

The use of antiVEGF agents (bevacizumab, Bev; Ziv-aflibercept and ramucirumab) in combination with second-line CT is supported by the positive results of several randomized phase III clinical trials^6–10^. Similarly, anti-EGFR agents (cetuximab, Cet, and panitumumab) have been shown to improve outcomes compared to CT alone in the second line setting in patients with wild type (WT) Kirsten rat sarcoma viral oncogene homolog (*KRAS*) mCRC^11–15^. Given the number of randomized trials demonstrating the efficacy of different second-line treatments in mCRC, strategy trials are needed to define the optimal use and best sequencing of all available agents.

**Figure 1:**
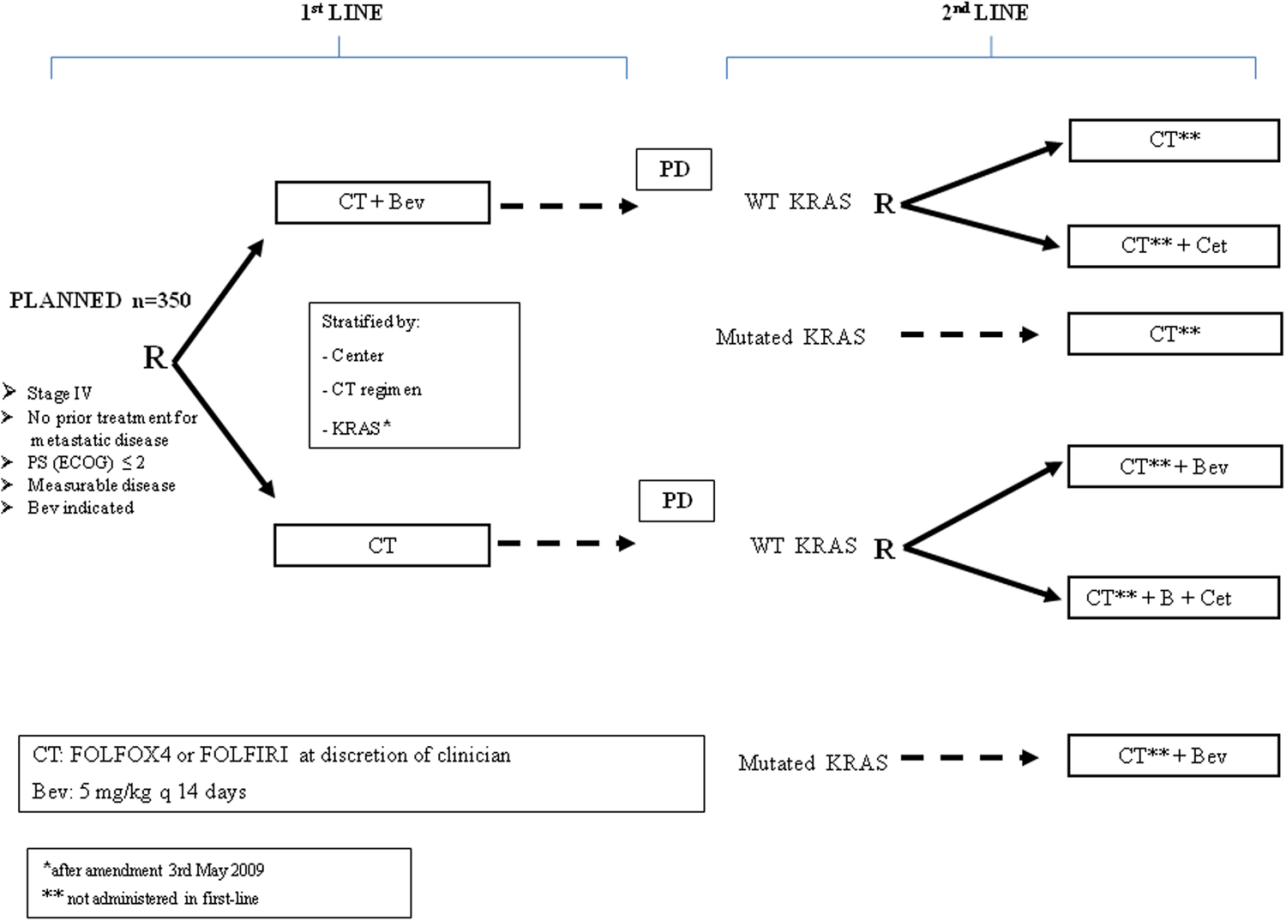
ITACa study design. R = randomized; PD = progressive disease; WT = wild type; MUT = mutated.

The ITACa trial, supported by the Italian Medicines Agency (AIFA), was designed to define the role of Cet and Bev in combination with standard chemotherapy (FOLFIRI or FOLFOX4) as first- and second-line treatment of mCRC (Fig. 1). Results from the first-line trial (Arm A: CT plus Bev vs Arm B: CT alone) were recently published and indicated no impact from the addition of Bev to standard CT in terms of PFS and OS^16^. The present paper reports the results of the second-line randomized studies, designed to determine the effect of adding Cet to standard CT or to CT plus Bev on progression-free survival (PFS). This study is registered on ClinicalTrials.gov (NCT01878422).

## Patients and Methods

### Study design

In the first-line trial, all eligible patients were randomized to receive either CT plus Bev (arm A) or CT alone (arm B). CT was FOLFIRI or FOLFOX4, at the discretion of the clinician, for either arm. Upon progression, all eligible patients could be randomized onto one of two independent second-line trials, Study 153 01/2A and 153 01/2B. In study 2A, arm A patients with WT KRAS were randomized to the other CT regimen (FOLFIRI or FOLFOX4) or the other CT plus Cet. Arm A patients with mutated KRAS were not randomized and were treated with the other CT regimen alone. In study 2B, arm B patients with wild type KRAS were randomized to the other CT plus Bev or the other CT plus Bev plus Cet. Arm B patients with mutated KRAS were not randomized and were treated with the other CT plus Bev (Fig. 1).

### Patient selection

Patients with at least one unidimensionally measurable lesion not amenable to curative resection, an Eastern Cooperative Oncology Group (ECOG) performance status of ≤2 (≤1 if aged ≥70 years), and an estimated life expectancy of at least 12 weeks were enrolled onto the trials. KRAS mutational status evaluation was mandatory after the protocol amendment no.1 of May 2009. Adequate hematologic, hepatic, and renal functions were required, as well as D-dimer within normal range (if abnormal, thrombembolic events had to be excluded). Chemoembolization and radical or cytoreductive or palliative surgery at the end of first-line therapy were allowed if there was evidence of progressive disease. Exclusion criteria included pregnancy or breast-feeding; clinically significant cardiovascular disease; known CNS metastases; interstitial pneumonia or extensive symptomatic fibrosis of the lungs; uncontrolled hypertension; pulmonary embolism or any arterial thromboembolism, deep vein thrombosis or other significant thromboembolic events, bleeding diathesis or coagulopathy, clinically significant peripheral vascular disease; chronic use of aspirin (>325 mg/day), anti-platelet agents or anticoagulants; proteinuria (if protein >30 mg/dL or + 1, ≤1 g of protein/24 h required).

The ITACa trial was approved by the local ethics committee (Comitato Etico Area Vasta Romagna) on September 19th, 2007 and was registered in our National Clinical Trials Observatory (Osservatorio delle Sperimentazioni Cliniche) and in the European Clinical Trials Database (EudraCT no. 2007–004539–44) before patient recruitment began. Registration on ClinicalTrials.gov (NCT01878422) was not mandate but was carried out at a later date (07/06/2013). All patients provided written informed consent and the studies were carried out in accordance with the Declaration of Helsinki under good clinical practice conditions and after full ethics committee approval of all participating centers (Comitato Etico Area Vasta Romagna e IRST, Comitato Etico Provinciale di Modena, Comitato Etico A.USL di Piacenza, Comitato Etico Interaziendale A.O.U. “Maggiore della Carità” di Novara, Comitato Etico Interaziendale dell’A.S.O. Santa Croce e Carle di Cuneo, Comitato Etico della Provincia di Modena, Comitato Etico della Provincia di Ferrara, Comitato Etico Unico per la Provincia di Parma, Comitato Etico Indipendente Azienda USL di Bologna, Comitato Etico della ASL LE di Lecce, Comitato Etico Provinciale di Belluno per la Sperimentazione Clinica).

### Treatment

The FOLFIRI and FOLFOX4 regimens were as previously described^3, 4^. Cet was administered on day 1 at an initial dose of 400 mg/m^2^ as a 2-hour intravenous infusion, and then at 250 mg/m^2^ intravenously over one hour on a weekly basis. Bev was administered after FU bolus as a 30- to 90-minute intravenous infusion at a dose of 5 mg/kg on day 1 if combined with CT alone, or on day 2 if combined with CT plus Cet. Treatment was to be continued until disease progression (PD), withdrawal of consent or unacceptable toxicity, whichever came first. Pre-specified dose modifications of CT were provided after the occurrence and resolution of severe hematologic or non-hematologic toxicity. No dose reductions of Bev were made in any patient. Bev was suspended in the event of grade 3 venous thromboembolism or incidentally discovered pulmonary embolus (until a stable dose of anticoagulant was administered), proteinuria >2 g protein/24 hours (until improvement to ≤2 g/24 hours), and uncontrolled hypertension. Bev was permanently discontinued in the presence of any grade 4 toxicity. The dose of Cet was reduced for recurrent Cet-related grade 3 toxicities only and at the second or third occurrence of grade 3 skin toxicity. Cet was discontinued after grade 3–4 allergic/hypersensitivity reactions, if more than two consecutive infusions were withheld or a fourth occurrence of a grade 3 skin toxicity occurred despite appropriate dose reduction. If one of the regimen components was discontinued due to toxicity, treatment could be continued with the remaining components.

### Outcomes

The primary objective was to determine, separately for each study, whether the addition of Cet to CT (Study 153 01/2A), or to CT plus Bev (Study 153 01/2B), would improve efficacy in terms of progression-free survival (PFS). The secondary objectives were objective response rate (ORR), overall survival (OS) and safety profile of the treatments administered.

PFS was defined as the time from the date of randomization in the second-line study to the date of the subsequent observation of documented PD or death due to any cause. Patients without PD at the time of analysis were censored at their last date of tumor evaluation. Patients undergoing curative metastasectomy were censored at the time of surgery. Response Evaluation Criteria in Solid Tumors (RECIST) guidelines were used to define all responses. An independent central review of patient scans was not carried out. OS was measured from the date of randomization in the 2^nd^ line studies to the date of death due to any cause or the last date the patient was known to be alive (censored observation). Adverse events were graded according to the National Cancer Institute Common Toxicity Criteria (NCI-CTC) for Adverse Events, Version 3.

### Statistical analysis

As previously mentioned^16^, the ITACa trial of first- and second-line management strategies was initially conceived to enrol 1000 candidates for first-line treatment to compare the efficacy of the two strategies in terms of overall survival. In the original design, hypothesizing that about 60% of the first-line population would be eligible for second-line studies, and that about 60% of patients would have a wild type KRAS tumor, we estimated that a population of about 400 patients (200/study) would be needed for the study to have sufficient power.

After the study was amended and the sample size of the first-line trial was reduced to 376 patients due to slower-than-expected patient recruitment, the primary endpoint of the first-line study became PFS and the statistical design of the second-line studies was also modified. According to literature data, the median time to progression after second-line treatment ranged from 4 to 6 months. Assuming a 48-month accrual period, a further 12-month follow-up and a 5% significance level (2-sided), we calculated that 80 patients randomized to each study would achieve an 80% power for PFS analysis under various assumptions of progression rate and true underlying reduction in HR by adding Cet to CT or to CT plus Bev.

Patients were randomized in a 1:1 allocation ratio. Separate randomization lists using a permuted block balanced procedure were generated for each participating center, stratified by the CT regimen and by *KRAS* status (WT/unknown or mutated, according to amendment no. 1 of 3^rd^ May 2009).

Safety analysis was based on the population of all treated patients (at least one cycle). Time to event data (PFS, OS) were described using Kaplan-Meier curves and compared by the logrank test (at a significance level of 5%). Ninety-five percent confidence intervals (95% CI) were calculated by non-parametric methods. Estimated hazard ratios (HRs) and their 95% CI were calculated using the Cox proportional-hazard model adjusted by center and CT regimen (FOLFOX4 or FOLFIRI). The ORR (complete response + partial response) was calculated with an exact 95% CI using standard methods based on binomial distribution. Non-interim efficacy analysis was planned. All p*-*values were based on two-sided testing and statistical analyses were performed using SAS statistical software version 9.4 (SAS Inc., Cary, NC).

### Data availability

All data generated or analyzed during this study are included in this published article (and its Supplementary Information files).

## Results

### Patients

**Figure 2:**
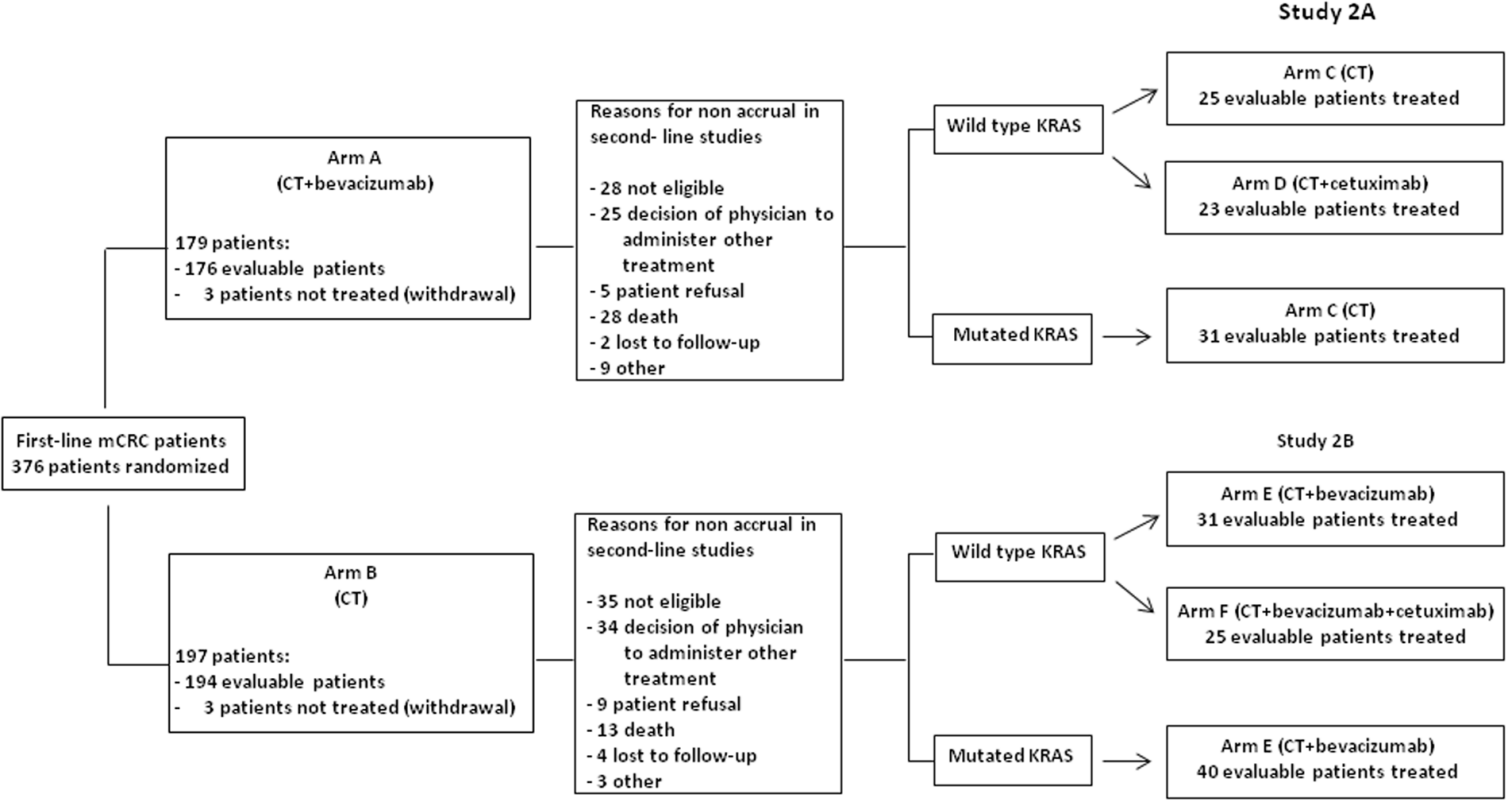
Flow-chart (CONSORT diagram). mCRC = metastatic colorectal cancer; ITT = intent-to-treat.

Between April 2008 and December 2015, 48 and 56 WT KRAS patients were randomized onto Studies 153 01/2A and 153 01/2B, respectively, while 31 and 40 KRAS mutated patients were treated without randomization in the former and latter study (Fig. 2). Demographic and clinical characteristics of 2A and 2B study patients were well balanced between treatment arms (Table 1).

**Table 1:** Baseline patient characteristics (KRAS WT).

Patient characteristics	Study 2A		Study 2B	
	CT(No = 25)*N* (%)	CT + Cet(No = 23)*N*(%)	CT + Bev(No = 31)*N* (%)	CT + Bev + Cet(No = 25)*N*(%)
Median age, years (range)	63 (44–82)	65 (35–82)	64 (45–81)	65 (33–79)
Gender				
Male	16 (64.0)	16 (69.6)	20 (64.5)	16 (64.0)
Female	9 (36.0)	7 (30.4)	11 (35.5)	9 (36.0)
Performance Status (ECOG)				
0	20 (80.0)	16 (69.6)	25 (83.3)	17 (68.0)
1–2	5 (20.0)	7 (30.4)	5 (16.7)	8 (32.0)
Tumor localization				
Rectum	8 (32.0)	3 (13.0)	10 (32.3)	4 (16.0)
Colon	17 (68.0)	20 (87.0)	21 (67.7)	21 (84.0)
Stage at diagnosis				
I-III	6 (24.0)	3 (13.6)	7 (23.3)	6 (25.0)
IV	19 (76.0)	19 (86.4)	23 (76.7)	18 (75.0)
CT regimen				
FOLFOX	9 (36.0)	9 (39.1)	15 (48.4)	10 (40.0)
FOLFIRI	16 (64.0)	14 (60.9)	16 (51.6)	15 (60.0)

CT, chemotherapy; Bev, bevacizumab; Cet, cetuximab.

### Treatment exposure

*Study 2A (WT KRAS patients)*. The median number of treatment cycles per patient was higher for the Cet and CT combination (8, range 3–13) than for CT alone (5, range 1–13), and the total number of treatment cycles was also superior in the experimental arm (219 vs. 144). Reductions in CT dose were more frequent in the CT plus Cet arm (34.7% vs. 24.3%), while cycle delays were comparable.

*Study 2B (WT KRAS patients)*. Although the patients in either treatment arm received a median of 6 cycles, the total number of treatment cycles was higher in the CT plus Bev group (352 vs. 263). Reductions in the CT dose were slightly more frequent in the CT plus Bev arm (20.2 vs. 12.6%), while cycle delays were comparable.

Treatment with Cet was provided for patients in the control arm of both studies after disease progression. A Cet-based third-line regimen was administered to 10/25 (40%) and 10/31 (32.3%) patients in studies 2A and 2B, respectively.

### Safety

Table 2 reports the most frequent adverse events observed in WT KRAS patients. Overall, the safety profile of the Cet-containing arms was consistent with prior studies, the only meaningful increase in toxicity observed regarding acneform rash. Unacceptable toxicity leading to treatment discontinuation occurred in 2 (8.7%) treated patients with CT plus Cet in study 2A (vs. 0 in CT alone arm) and in 1 (4%) treated with CT plus Cet plus Bev in study 2B (vs. 3.2% in the CT + Bev arm). Only one toxic death (due to febrile neutropenia) was reported in the study 2A CT plus Cet arm.

**Table 2:** Toxicity (KRAS WT).

	CT		CT + Cet		CT + Bev		CT + Bev + Cet	
	*N* (%)		*N* (%)		*N* (%)		*N* (%)	
	Any grade	G3-G4	Any grade	G3-G4	Any grade	G3-G4	Any grade	G3-G4
Nausea	8 (32.0)	1 (4.0)	4 (17.4)	0	10 (32.3)	0	4 (16.0)	2 (8.0)
Vomiting	10 (40.0)	0	4 (17.4)	0	6 (19.3)	0	6 (24.0)	2 (8.0)
Constipation	6 (24.0)	1 (4.0)	3 (13.0)	0	6 (19.3)	0	6 (24.0)	1 (4.0)
Diarrhea	8 (32.0)	1 (4.0)	12 (52.1)	0	14 (45.1)	1 (3.2)	11 (44.0)	4 (16.0)
Stomatitis	2 (8.0)	0	8 (34.8)	2 (8.7)	10 (32.2)	2 (6.4)	5 (20.0)	1 (4.0)
Fatigue	7 (28.0)	3 (12.0)	14 (60.8)	5 (21.7)	10 (32.2)	2 (6.4)	11 (44.0)	1 (4.0)
Fever	10 (40.0)	0	12 (52.1)	1 (4.3)	7 (22.5)	0	9 (36.0)	0
Skin	1 (4.0)	1 (4.0)	15 (65.1)	5 (21.7)	2 (6.4)	0	13 (52.0)	3 (12.0)
Pain	9 (36.0)	2 (8.0)	10 (43.4)	1 (4.3)	14 (45.1)	1 (3.2)	13 (52.0)	1 (4.0)
Respiratory system	1 (4.0)	0	2 (8.6)	2 (8.6)	5 (16.1)	1 (3.2)	4 (16.0)	0
Neurologic system	6 (24.0)	1 (4.0)	3 (13.0)	0	12 (38.6)	2 (6.4)	6 (24.0)	2 (8.0)
Anemia	3 (12.0)	0	4 (17.3)	2 (8.7)	4 (12.8)	1 (3.2)	4 (16.0)	1 (4.0)
Leukopenia	7 (28.0)	2 (8.0)	12 (52.1)	1 (4.3)	10 (32.2)	2 (6.4)	5 (20.0)	2 (8.0)
Neutropenia	13 (52.0)	10 (40.0)	17 (73.9)	12 (52.2)	18 (57.9)	15 (48.3)	8 (32.0)	8 (32.0)
Thrombocytopenia	6 (24.0)	1 (4.0)	5 (21.7)	0	6 (19.3)	0	4 (16.0)	2 (8.0)
Febrile neutropenia	0	0	1 (4.3)	1 (4.3)	2 (6.4)	2 (6.4)	0	0
Infection	0	0	3 (12.9)	1 (4.3)	0	0	4 (16.0)	1 (4.0)
Hemorrhage	1 (4.0)	0	1 (4.3)	0	5 (16.1)	0	3 (12.0)	1 (4.0)
Hypertension	2 (8.0)	0	2 (8.6)	0	6 (19.3)	1 (3.2)	4 (16.0)	1 (4.0)
Proteinuria	3 (12.0)	0	4 (17.3)	0	8 (25.7)	0	5 (20.0)	0
Thrombosis	4 (16.0)	1 (4.0)	1 (4.3)	0	2 (6.4)	2 (6.4)	4 (16.0)	2 (8.0)

CT, chemotherapy; Cet, cetuximab; Bev, bevacizumab.

### Efficacy

The data cut-off for efficacy analysis of both studies was April 2016 when the median duration of follow-up was 46 months (range 1–61 months).

#### Study 2A (WT KRAS patients)

**Figure 3:**
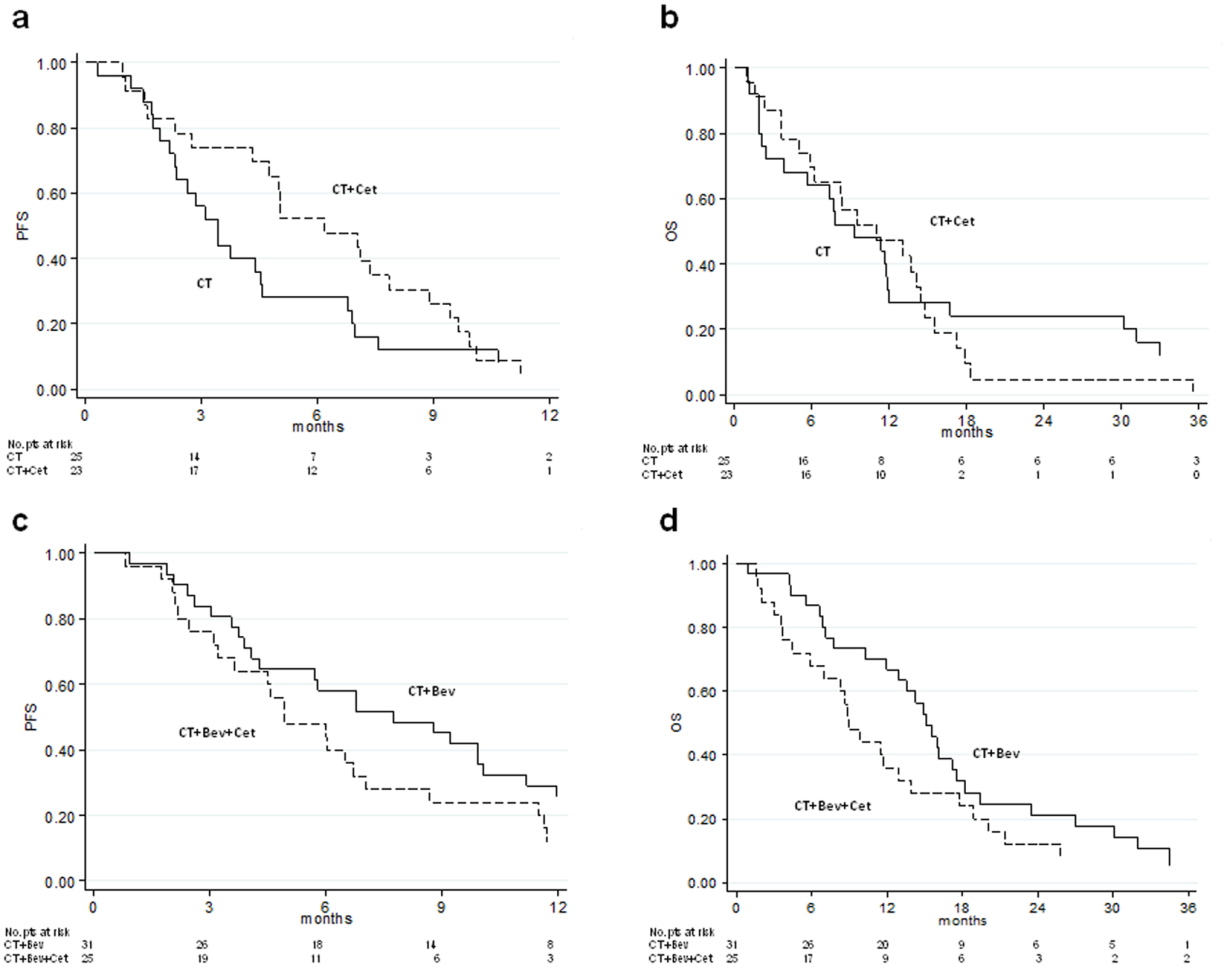
Kaplan-Meier estimate of progression-free survival of Study 2A (**a**), overall survival of Study 2A (**b**), progression-free survival of Study 2B (**c**) and overall survival of Study 2B (**d**). PFS = progression-free survival; HR = hazard ratio; OS = overall survival.

At the data cut-off, 48 (100%) events had occurred for PFS. All patients had progressed or died. Median PFS was 3.4 (95% CI 2.3–4.6) and 6.2 (95% CI 4.3–7.8) months for the CT and CT plus Cet arms, respectively, with a HR of 0.64 (95% CI 0.35–1.16, p = 0.144) (Fig. 3a) adjusted by center and CT regimen (FOLFOX4 or FOLFIRI).

Overall, 45 patients died, 23 (92%) in the CT arm and 22 (95.7%) in the CT plus Cet arm. Median OS was 9.3 (95% CI 3.8–12.0) and 11.1 (95% CI 5.9–14.4) months in the CT and CT plus Cet groups, respectively, with a HR of 1.30 (95% CI 0.70–2.44, p = 0.402) (Fig. 3b) adjusted by center and CT regimen (FOLFOX4 or FOLFIRI). The ORR for CT plus Cet was 30.5% compared to 16% for CT alone (p = 0.398).

#### Study 2B (WT KRAS patients)

At the data cut-off, 55 (98.2%) events had occurred for PFS. Median PFS was 7.7 (95% CI 4.1–10.1) and 4.9 (95% CI 3.2–7.0) months for the CT plus Bev and CT plus Cet plus Bev arms, respectively, with a HR of 1.31 (95% CI 0.76–2.26, p = 0.330) (Fig. 3c) adjusted by center and CT regimen (FOLFOX4 or FOLFIRI).

Overall, 51 patients died, 28 (90.3%) in the CT plus Bev arm and 23 (92%) in the CT plus Cet plus Bev arm. Median OS was 15.1 (95% CI 12.0–17.5) and 8.9 (95% CI 5.9–13.9) months for the CT plus Bev and CT plus Cet plus Bev groups, respectively, with a HR of 1.31 (95% CI 0.73–2.35, p = 0.361) (Fig. 3d) adjusted by center and CT regimen (FOLFOX4 or FOLFIRI). The ORR for CT plus Bev was 32.2% compared to 16% for CT plus Cet plus Bev (p = 0.277).

#### Mutated KRAS patients

At the data cut-off, all patients in the study 2A with mutated KRAS had progressed and 30 (96.8%) had died. Median PFS was 4.2 months (95% CI 2.4–7.9), median OS was 8.7 months (95% CI 5.3–12.9) and ORR was 25.8%. 39 (97.5%) events for PFS and 37 (92.5%) for OS were reported among study 2B patients with mutated KRAS. Median PFS was 8.6 months (95% CI 6.8–11.2), median OS was 15.1 months (95% CI 12.9–18.0) and ORR was 25%.

#### Subgroup analyses

In the light of recent literature data, patients with *WT KRAS* were further evaluated for mutations in NRAS and BRAF and in other *KRAS* exons. Among 2A patients, 7 RAS mutations and 7 BRAF mutations were detected, while 8 RAS mutations and 3 BRAF mutations were detected among 2B patients.

#### Study 2A (all RAS WT patients, regardless of BRAF status)

Median PFS was 7.0 months (95% CI 2.7–8.9) and 3.3 (95% CI 1.9–4.6) months for CT plus Cet and CT, respectively, with an HR of 0.56 (95% CI 0.29–1.09, p = 0.088) adjusted by center and CT regimen (FOLFOX4 or FOLFIRI). Median OS was 11.1 (95% CI 5.0–14.4) in the CT plus Cet arm and 7.8 (95% CI 1.9–16.7) in the CT alone arm, with an adjusted HR of 1.27 (95% CI 0.64–2.50, p = 0.489).

#### Study 2A (all RAS/BRAF WT patients)

Median PFS was 7.5 months (95% CI 4.3–9.6) and 3.4 months (95% CI 2.3–4.6) for CT plus Cet and CT, respectively, with an adjusted HR of 0.48 (95% CI 0.23–0.99, p = 0.049). Median OS was 13.1 months (95% CI 5.0–14.4) in the CT plus Cet arm and 9.6 months (95% CI 1.9–16.7) in the CT alone arm, with an adjusted HR of 1.29 (95% CI 0.61–2.74, p = 0.503).

#### Study 2B (all RAS WT patients, regardless of BRAF status)

Median PFS was 6.0 months (95% CI 3.7–8.7) and 7.3 months (95% CI 4.1–9.9) for CT plus Cet plus Bev and CT plus Bev, respectively, with an HR of 1.04 (95% CI 0.56–1.95, p = 0.892) adjusted by center and CT regimen (FOLFOX4 or FOLFIRI). Median OS was 10.7 months (95% CI 4.5–17.8) in the CT plus Cet plus Bev arm and 15.1 months (95% CI 10.3–17.2) in the CT plus Bev arm, with an adjusted HR of 1.03 (95% CI 0.52–2.04, p = 0.927).

#### Study 2B (all RAS/BRAF WT patients)

Median PFS was 6.0 months (95% CI 3.2–8.7) and 7.3 months (95% CI 4.1–9.9) for CT plus Cet plus Bev and CT plus Bev, respectively, with an adjusted HR of 1.19 (95% CI 0.60–2.37, p = 0.609). Median OS was 11.5 months (95% CI 3.7–17.8) in the CT plus Cet plus Bev arm and 15.1 months (95% CI 10.3–17.2) in the CT plus Bev arm, with an adjusted HR of 1.20 (95% CI 0.57–2.52, p = 0.629).

## Discussion

The ITACa second-line trial is, to our knowledge, the first randomized trial to prospectively investigate the role of cetuximab in a WT KRAS population in combination with second-line CT (Study 2A) or CT plus Bev (Study 2B). Since 2008, the use of anti-EGFR agents has been restricted to WT KRAS patients as it has been shown that KRAS exon 2-mutated tumors are resistant to this therapy^17^. Recently, several studies investigating the predictive and prognostic role of RAS mutations other than in KRAS exon 2 reported that patients with these mutations did not respond to therapy. Consequently the use of anti-EGFR drugs has been further limited to all WT RAS tumors^18^.

The study 2A compared the activity of Cet plus second-line CT with that of CT alone. Our results suggest that Cet improved PFS by almost 3 months and OS by almost 2 months, in addition to doubling ORR. Although median OS was higher in the CT plus Cet arm than in the CT arm, the HR shows an increased risk of death for the CT plus Cet arm due to the longer survival of CT arm patients after the 15-month time point (Fig. 3b). A possible explanation for this could be the favorable outcome of patients receiving a Cet-based third-line regimen. The small sample size prevents us from evaluating these valid results from a statistical point of view. The subgroup analysis of all *RAS/BRAF WT* patients showed a significant improvement in PFS in the experimental arm (HR 0.48, 95% CI 0.23–0.99, p = 0.049).

The efficacy of Cet in combination with CT after first-line treatment failure was investigated in the multicenter, open-label, phase III EPIC trial in which 1298 patients unselected for KRAS were randomly assigned to Cet plus irinotecan or irinotecan alone. The median OS (primary endpoint) was similar in both arms: 10.7 and 10.0 months for CT plus Cet and CT alone, respectively (HR = 0.975; p = 0.71). However, CT significantly improved PFS (median, 4.0 vs. 2.6 months; HR = 0.692; p ≤ 0.0001) and ORR (16.4% vs. 4.2%; p < 0.0001) in the intention-to-treat population^11^. No differences in terms of OS were seen in a subsequent analysis of a small subset of patients with WT KRAS tumors (15% of the entire population)^12^. Similar results were observed in a randomized phase III study of second-line panitumumab in combination with FOLFIRI compared to FOLFIRI alone in patients with metastatic colorectal cancer. A significant improvement in PFS was observed in the WT KRAS subpopulation when panitumumab was added to CT (5.9 vs. 3.9 months, HR = 0.73; p = 0.004), together with a non-significant trend towards increased OS (14.5 months vs. 12.5 months, HR = 0.85, p = 0.12)^13^.

A ‘head to head’ comparison between VEGF and EGFR inhibitors in combination with second-line chemotherapy should be considered in future randomized trials as this would help to identify the best second-line regimen in the RAS WT population. The study 2B investigated the role of Cet added to second-line CT plus Bev. Two large randomized phase 3 trials investigated the efficacy and safety of treatment regimens incorporating CT in combination with Bev and an EGFR inhibitor: the Panitumumab Advanced Colorectal Cancer Evaluation (PACCE) study analyzed CT plus Bev with or without panitumumab, while the CAIRO2 study assessed the efficacy and safety of capecitabine plus oxaliplatin and Bev with or without Cet. In either trial, the combination of the 2 monoclonal antibodies was associated with increased toxicity and decreased efficacy^19, 20^. The ITACa trial was ongoing when the results from both studies were presented and the Steering Committee evaluated the idea of amending the study by eliminating the CT plus Bev plus Cet arm. However, following a review of available literature data, it was decided not to proceed with any modification because both PACCE and CAIRO2 trials were conducted before KRAS mutations were identified as a predictor of poor response to anti-EGFR monoclonal antibodies, therefore patients were not selected. Moreover, both were first-line trials and the CT regimens analyzed differed from those of the ITACa trial (panitumumab in PACCE instead of Cet and capecitabine in CAIRO2 instead of 5FU). All ITACa investigators were nonetheless instructed to monitor carefully for toxicity. Our results of a reduction of almost 3 months in PFS and a 50% decrease in median OS and ORR would appear to confirm the detrimental effect of adding Cet to CT plus Bev, even in WT KRAS patients.

In conclusion, our findings indicate that the addition of Cet to second-line CT increased PFS in mCRC patients with WT KRAS, whereas the addition of Cet to CT + Bev was associated with shorter PFS. Whilst we are aware that the small population of both second-line studies does not enable definitive conclusions to be drawn, the clinical relevance of our results is high. The novelty of the work stems from the fact that it is a follow-on of a study in which first- and second-line strategies were jointly evaluated and all patients received the same first-line treatment, thus limiting the biases due to possible interactions, resistance or long-term effects that can occur when the study population has received heterogeneous treatments.
